# Taking the Big Leap | understanding, accessing and improving behavioural science interventions

**DOI:** 10.3389/fpubh.2024.1355539

**Published:** 2024-08-07

**Authors:** Nishan Gantayat, Anushka Ashok, Pallavi Manchi, Rosemary Pierce-Messick, Rahul Porwal, Alok Gangaramany

**Affiliations:** Final Mile Consulting, New York, NY, United States

**Keywords:** public health, behavioural science, HIV prevention, behavioural economics, TB, sustainability, policy

## Abstract

Applied behaviour science’s focus on individual-level behaviours has led to overestimation of and reliance on biases and heuristics in understanding behaviour and behaviour change. Behaviour-change interventions experience difficulties such as effect sizes, validity, scale-up, and long-term sustainability. One such area where we need to re-examine underlying assumptions for behavioural interventions in Human Immunodeficiency Virus (HIV) and Tuberculosis (TB) prevention, which seek population-level benefits and sustained, measurable impact. This requires taking a “Big Leap.” In our view, taking the big leap refers to using a behavioural science-informed approach to overcome the chasms due to misaligned assumptions, tunnel focus, and overweighting immediate benefits, which can limit the effectiveness and efficiency of public health programmes and interventions. Crossing these chasms means that decision-makers should develop a system of interventions, promote end-user agency, build choice infrastructure, embrace heterogeneity, recognise social and temporal dynamics, and champion sustainability. Taking the big leap toward a more holistic approach means that policymakers, programme planners, and funding bodies should “Ask” pertinent questions to evaluate interventions to ensure they are well informed and designed.

## 1 Introduction

Despite remarkable advancements in HIV treatment accessibility, preventing new infections remains crucial in ending the HIV pandemic (1). Though a wider basket of prevention products is available, many individuals who would benefit hesitate to use them. This challenge is not unique to HIV; it reflects broader and often interconnected individual, social, and structural level issues hindering access, uptake, and sustainable behaviour change across domains like financial inclusion, water & sanitation, etc., due to inadequate consideration of complex factors influencing human behaviour.

Behavioural science attempts to address these challenges by understanding the preferences and drivers of behaviour change related to the uptake, initiation, maintenance, and persistence of prevention products and services. It involves the systematic study of observable actions and mental phenomena such as knowledge, attitudes, beliefs, motivations, perceptions, cognitions, and emotions. Drawing from psychology, cognitive science, social science, and economics, behavioural science examines the interplay between human traits and contextual factors that influence behaviour (2, 3).

In the past two decades, behavioural science has seen shifts. It has moved from academic settings to practical applications impacting health and well-being, as noted in strategic guidance documents by PEPFAR, USAID (4), WHO, and UN (5–8). Applied behavioural science has become influential in informing policy-making and organisational strategies, emphasisng behaviour change in addition to theoretical understanding basic human behaviours. This has led to the development of behavioural insights to inform policymakers about human behaviours driving economic and societal outcomes (9–11).

Nevertheless, the rise of behavioural science and behavioural insights has sparked scrutiny and scepticism. Concerns have emerged regarding the rigour of empirical methods, replicability, scalability, and the long-term sustainability of their intervention results (12, 13). Early successes in behavioural interventions have shown promise and success in controlled settings but yielded mixed results when scaled up in other locations, raising questions about their external validity (14–16). Additionally, social determinants of behaviour are time-variant and can change due to shifting environmental factors, including technological advancements, and thus necessitate the reassessment of behavioural strategies.

This perspective aims to advocate for fundamental changes in the application of Behavioural science, bringing into focus some of the limitations of behavioural insights, and the approach it currently undertakes to produce desired behaviour change. We use HIV/TB prevention as the area of focus to elucidate this perspective. We present barriers in the form of “Chasms” that represent limitations in the current approach. We then highlight some tenets that form the “Big Leap” required to cross the chasms and advance behavioural science in public health. For decision-makers, we provide a checklist of “Asks” that can help them evaluate if a given intervention(s) will enable them to take the big leap.

## 2 Chasms: gaps in applying behavioural science to interventions

Initially, the use of behavioural science, notably in behavioural economics, aimed to comprehend the intricacies of human behaviour affecting decision-making under uncertainty (17–19). In the context of HIV/TB this meant understanding the adoption of HIV prevention methods like condoms or accessing HIV treatment for People Living with HIV (PLHIV) (20). However this application of behavioural science, particularly in behavioural economics, has been limited to exploring biases, heuristics, and the concept of a rational individual driven by incentives (21). Aside from the proliferation and replication problems, this has led to ill-informed and/or interventions with limited success.

Looking at the current landscape of interventions informed by behavioural science, there appear to be 6 broader limitations that prevent us from effectively using behavioural science for the desired goals.

### 2.1 Individuals over interactions: behavioural insights are too focused on the individual, not considering the interactions

Behavioural science often focuses on the individual, examining their judgement, decisions, and the potential for prediction and control. However, this narrow perspective fails to consider that behaviours emerge as a result of continuous and often simultaneous interactions occurring in an individual’s ecosystem (22). Programmes fail to consider the significance of individuals’ multiple identities that exist such as their roles as friends and partners, and how these identities shape their decisions (23).

In TB prevention interventions, including reminders, tracking, etc., an excessive focus on individual behavioural traits has resulted in a disregard for the unique dynamics of families and other social institutions within which decisions are made (24–27).

### 2.2 Assumption of static context: behavioural insights ignore the dynamic nature of the world in which people live

Complexities of human behaviour, embedded in an understanding of a dynamic environment, evolving individual (e.g., risk perception), social (e.g., marriage), and structural (e.g., policies and laws) determinants, that are continuously shaped by individual and collective actions over time are often overlooked in solution design (7, 28, 29). For instance, addressing HIV prevention among Adolescent Girls and Young Women (AGYW) requires recognising the changing nature of their contexts beyond the education system, and includes evolving psycho-social and physiological developments shaped by biological, social, and economic vulnerabilities (30–32).

For instance, reminders as intervention ignore patients’ evolving risk perceptions tied to their changing physiological state, i.e., an improved physiological condition leads to lower risk perception, whilst overexposure to risk messaging diminishes risk saliency (33, 34). The effect of such interventions on treatment adherence in TB patients have been seen to wane after 6–8 weeks (35–37).

### 2.3 One size fits all: behavioural insights frequently ignore the heterogeneity in the population

Behavioural insights are typically derived from research conducted in controlled academic settings and by using samples suited to research design and not representative enough for the findings not to be generalisable for the broader population. Methods such as RCTs assume a certain level of homogeneity within target populations and fail to take into consideration the diverse needs and identities that are generated and/or co-exist within the same population (10, 38).

In India, an intervention deploying peer mobilisers to reach Men who have Sex with other Men (MSM) online for HIV Testing (HIVT) overlooked varying health-seeking attitudes within MSM sub-populations and resulted in lower engagement, low-risk saliency, and impacted HIVT service uptake (39).

### 2.4 Ignoring second-order effects: behavioural interventions frequently ignore the long-term and indirect effects of interventions

Nudges are a type of intervention that steers individuals toward the desired action. They can be effective in specific and well-defined problems. However, their success is limited to short-term outcomes. A nudge-based approach considers that behaviour change occurs in isolation without disrupting the environment around it or having spillover effects (40, 41).

Interventions often fail to look at stigma in terms of the second-order effect generated by an intervention. Scale-up of Differentiated Service Delivery (DSD) models to include Pre-exposure Prophylaxis (PrEP), has raised the challenge of the possibility of conflation of PrEP with ART (42). In South Africa, a peer-delivered ‘Undetectable = Untransmittable’ (U = U) message improved men’s HIV testing uptake but did not consider the possibility of experiencing stigma (43).

In an incentive-based solution study conducted in Tanzania, economic incentives such as transport vouchers improved immediate willingness to take up Voluntary Medical Male Circumcision (VMMC) but did not capture if the interventions led to linkage to care (44).

### 2.5 Focused on the observables: behavioural insights frequently miss out on the unarticulated needs of users

Cold affective state, which reflects emotional detachment or indifference in user behaviour best captures what users can articulate in the moment. However, risks and motivations are typically transient, varying with affect (45–47). It is important to capture and understand them in a hot state, i.e., an emotionally charged or heightened state of behaviour. Solutions assume elements such as risks and emotions, that are involved in decision-making, to be constant. But in real-time, they are felt in spikes and troughs. Interventions often target behaviours in a “cold state,” where users deliberate on risks and choose an action. However, in the decision context, users are in a hot state where individuals use mental shortcuts and emotional cues to assess risks and end up choosing a different course of action.

Recently, an HIV intervention tool has been developed to help users navigate to an appropriate product for HIV prevention. It asks a series of behaviour-centred questions to situate users within a particular need and guides them to reach the right product. But this kind of systematic thinking may be too cognitively burdening whilst an individual is about to enter an aroused state, like sexual activity (48–50). But in real-time, users might not end up taking the right product even if they have been guided to one by the tool.

### 2.6 Issue with irrationality: assumption that all users are irrational leads to poor empathy

Behavioural science, often associated with the concept of the “myth of human rationality” drives behaviour-change interventions by exploring biases users face whilst making decisions. Equating sub-optimal decisions or divergent behaviours of individuals to biases or lack of information can lead to underestimating them and limiting our understanding of how they manifest, thereby not achieving user-centric interventions. The decision-making pathways and underlying emotions that contribute to the observed outcomes are often under-emphasised and produce a partial understanding of the influences that determine behaviour.

## 3 Big Leap: advancing behavioural science in public health interventions

Given the drawbacks in the current approach to the application of behavioural science, we propose the following shifts to achieve the ‘big leap’ to advance the practise of behavioural science and to improve the impact and sustainability (financial and benefits) of public health interventions.

### 3.1 Develop a system of interventions

*Behavioural science-informed approach should not limit itself to individual interventions and nudges, but embrace a multi-level approach towards creating a collective set of interventions that drive behaviour change*.

Rarely has a single intervention been seen to be working or producing sustained benefits. Solutions like incentives or vouchers for HIVT among MSM, have not been able to showcase repeated behaviours. To drive behaviour change, there is a need to strive for interventions at different touch points involving various agents and systems, and the different needs of the users.

- *In Botswana, Prevention of Mother-To-Child Transmission (PMTCT) interventions have been multilateral and holistic, involving all levels of care (such as facility, community, etc.), and different actors (such as mothers, Healthcare Workers (HCWs), Community Health Workers CHWs), etc.). By doing so, PMTCT programs have been able to leverage the contextual identity of the mothers involved and their motivation of being “a responsible mother who wants to keep her child healthy”* (51).
- *Combining interventions like personnel and peer training, active case finding, patient counselling, onsite sputum collection, and expedited treatment initiation, have been effective in improving case detection and treatment outcomes for TB. Such an approach shifts the onus of responsibility from patients to the healthcare system which, in turn, helps to reduce the cognitive load associated with expected discomfort, and anticipated stigma* (52).

### 3.2 Promote end-user agency

*Behaviour change interventions addressing individual-level biases and heuristics should consider preserving users’ control over their choices.*

Restricting the behavioural understanding to biases and mental models reduces behavioural drivers to an individual level and can lead to ignorance of the ecosystem surrounding the behaviour. It is therefore important to evolve an understanding of behaviours in terms of decision-making mechanisms, decision levers, and interactions where they manifest, all whilst providing effective choice to users whilst attempting to manipulate their choices to address biases.

- *In an intervention deployed in the emergency ward of a hospital to improve HIV testing acceptance, active choice was given to users where instead of an opt-in or opt-out option, patients were asked if they would want to get tested after providing them with the necessary information. This active choice treatment arm showed relatively higher acceptance for HIV testing as compared to opt-in but lower than the opt-out option* (53).

### 3.3 Build choice infrastructure

*Behaviour change interventions that focus on altering immediate decision environments should be supplemented with a deeper understanding of underlying infrastructure,* i.e.*, processes and structures.*

Instead of focusing on choice architecture, which relies on recognition of contextual information and the influence of presenting relevant information, choice infrastructure that establishes foundational systems and structures that facilitate decision-making should be prioritized (54). For example, combining micro-environmental nudges such as prompting, framing, and feedback in communications with system/structural level changes (55, 56)

- *In Mozambique, a Combination Intervention Strategy (CIS) intervention included point-of-care CD4 testing at the time of diagnosis, accelerated ART initiation, and Short Message Service (SMS) health messages and reminders, along with non-monetary vouchers. The CIS offered a more comprehensive set of services that made it easier for the patients to take preventive action* (57).

### 3.4 Embrace heterogeneity

*Behavioural understanding of user behaviours should consider the sub-populations and the diverse set of needs that exist within these sub-populations.*

Public health can limit itself if needs are narrowly defined, such as thinking of HIV prevention in terms of sexual behaviours. There is a requirement to move toward a broader set of needs of beneficiaries to drive the development of products and programmes.

- *Peer outreach to identify hotspots and subpopulation clusters has led to effective micro-planning interventions. Peers as messengers create relevance for the services/products and also help tackle the ‘perceived’ ill intent of many public health programs thus meeting the different safety and risk management needs of the sub-populations* (58, 59).

### 3.5 Recognise social and temporal dynamics

*Behaviour change interventions should build for non-linear and evolving user dynamics.*

Users live in a dynamic environment where certain interactions and events can alter their needs and preferences. In addition, life course changes (e.g., school dropouts, marriage, childbirth, etc.) can also impact their preferences for products and services. Public health programme delivery may also evolve due to sudden events such as a new pandemic. As such, there is a need to design interventions that can withstand such dynamic changes.

- *In response to COVID-19, national ministries of health adapted HIV service delivery guidelines to ensure uninterrupted access to ART, and to limit the frequency of contact with health facilities. This included expanding the eligibility of DSD for HIV treatment, extending multi-month dispensing (MMD), reducing the frequency of clinical consultations, and encouraging community-based models* (60).

### 3.6 Champion sustainability

*Behaviour change interventions should take into consideration the long-term financial viability and second-order effects of the expected change.*

For domains like public health where behaviours surround a public good, it is important to focus both on long-term change and sustainability which include ensuring interventions understand resource availability and transition toward domestic ownership of the programmes. It is important to consider the spillover effects of the intervention. E.g., what would happen after the incentives are removed? Will the interventions be feasible once the government takes over the programme?

## 4 The asks: rethinking behavioural science-led interventions

For taking the “Big Leap” and integrating behavioural science-informed research for effective and efficient programming, we outline 6 guiding questions that policymakers and funding organisations can pose whilst assessing and supporting interventions:

- *Does the intervention involve and influence multiple actors and interactions between actors?*
  Interventions should account for the intricate systems surrounding a target behaviour and its users. Example: Single-behaviour correction interventions may fall short of achieving lasting impact.
- *Does the intervention go beyond simplistic assumptions about “irrationality,” biases, and heuristics?*
  Interventions should delve into the complexities of human behaviour rather than merely addressing biases and heuristics. Example: Relying solely on reminders to tackle adherence challenges related to treatment regimens may offer limited effectiveness.
- *Does the intervention rely on more than choice manipulation tactics* (e.g.*, opt-in/defaults*)*?*
  Interventions employing behavioural “tricks” to enforce user actions can erode long-term trust and sustained benefits. Example: Making participation a default option to boost programme enrolment may not lead to enduring impact and consistent engagement.
- *Does the intervention consider its impact within the social context of the user?*
  Interventions should explore how changes in user actions affect their social settings. Example: An increase in HIV prevention uptake can intensify stigma within the user community, potentially yielding adverse results on effective use.
- *Does the intervention acknowledge the dynamic and heterogeneous nature of user goals, contexts, and needs?*
  Interventions should recognise the diversity within populations, comprising sub-populations operating in distinct contexts. Example: Assuming all Adolescent Girls and Young Women (AGYWs) have uniform goals may fail, as they have different relationships and sexual health objectives. A one-size-fits-all approach may prove inadequate.
- *Does the intervention have a medium to long-term outlook on viability and the sustainability of benefits?*
  Interventions should be financially sustainable in the long run, even as funding for programmes or interventions evolves. Example: Programmes that incentivise users through vouchers may not be economically viable in the long term.

## 5 Conclusion

**Figure 1:**
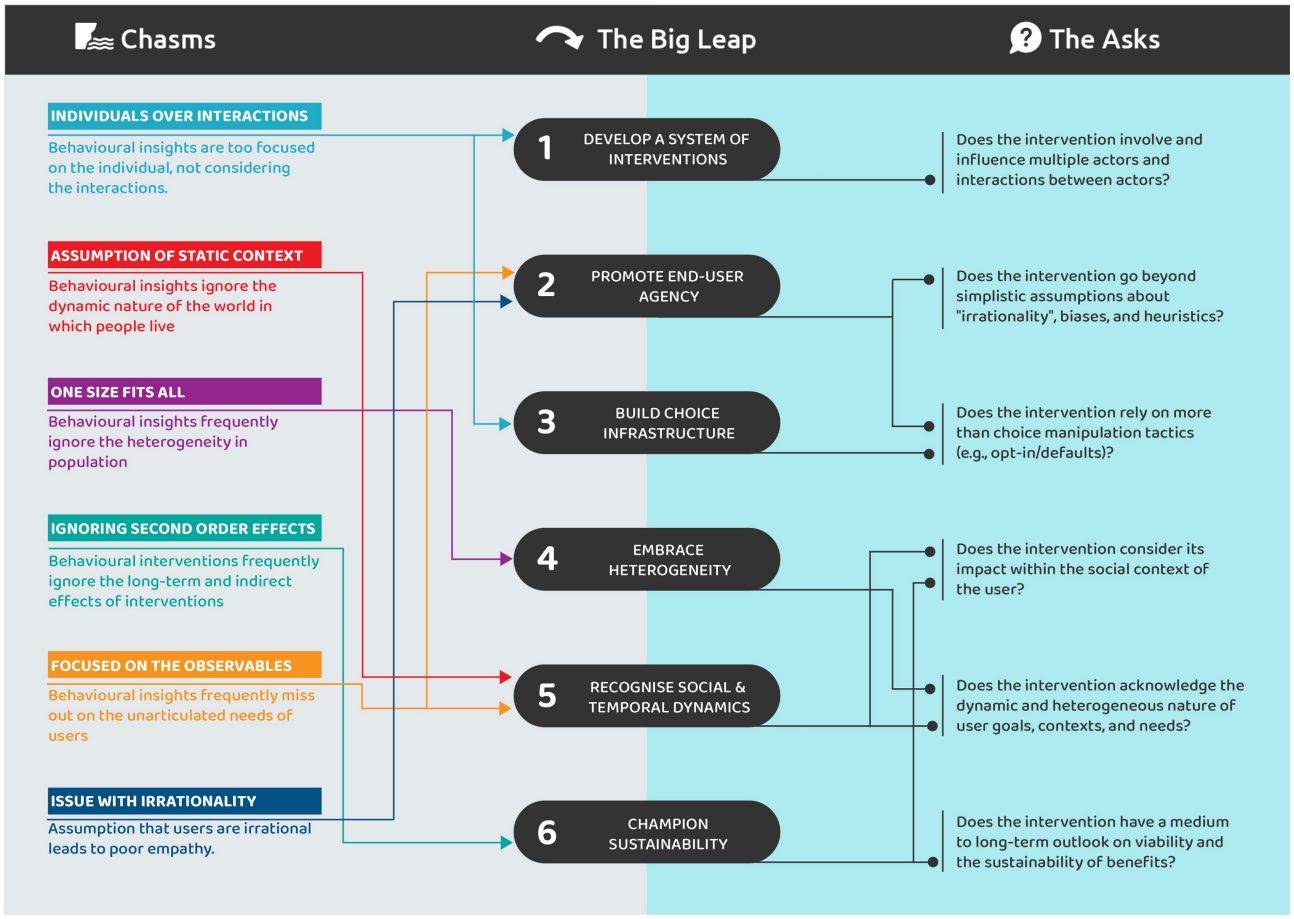
A snapshot view of the chasms encountered in application of behavioural science, the big leap that is required to cross the chasms and the asks to rethink behavioural interventions.

Behavioural science has become important to development programmes, including the use of nudges and choice architecture to promote healthy behaviours. However, the widespread application of behavioural science as a panacea for all challenges comes with its own set of limitations. As depicted in Figure 1, chasms exist that hinder the implementation of behavioural science-informed interventions. This collective questioning of the capacity of behavioural interventions to meaningfully contribute to programmatic and policy goals is justified particularly in domains such as public health. In public health, achieving long-term, substantial behavioural transformation is essential, making it imperative to establish accurate behavioural mechanisms for lasting and large-scale impact, especially amid funding constraints (61–65).

Bridging them requires taking a big leap forward. To address these challenges, where sustained and integrated behavioural change is crucial, it is imperative to establish accurate behavioural mechanisms for lasting and large-scale impact, especially amid funding constraints (66).

This paper and framework describe a principled approach to thinking about the complexities influencing behavioural change and maintenance in real-world settings. The themes identified aim to address a spectrum of challenges that hinder programme efficacy. Given the future HIV prevention efforts and investments including new products such as long-acting injectable cabotegravir (CAB-LA), it is useful to think about this framework in terms of how it needs to be applied to drive investment decisions. The goal is to help funding bodies and donors to effectively understand, assess and improve behavioural science-informed interventions for sustained benefits. This can support programmes to identify goals, conduct gap analysis, iterate and monitor interventions, and then sustain and scale-up interventions. Behavioural science practitioners must adopt user-centric approaches, recognising that health behaviour originates from the intricate interactions of individual, social, and structural factors.

## Data availability statement

The original contributions presented in the study are included in the article/supplementary material, further inquiries can be directed to the corresponding author.

## Author contributions

NG: Conceptualization, Data curation, Formal analysis, Investigation, Methodology, Project administration, Resources, Supervision, Validation, Visualization, Writing – original draft, Writing – review & editing. AA: Conceptualization, Formal analysis, Methodology, Supervision, Validation, Visualization, Writing – review & editing. PM: Conceptualization, Formal analysis, Methodology, Resources, Supervision, Validation, Visualization, Writing – review & editing. RP-M: Data curation, Formal analysis, Investigation, Resources, Supervision, Validation, Writing – review & editing. RP: Data curation, Investigation, Methodology, Resources, Validation, Visualization, Writing – original draft, Writing – review & editing. AG: Conceptualization, Formal analysis, Methodology, Project administration, Supervision, Validation, Visualization, Writing – review & editing.
